# BMI Modulates Calorie-Dependent Dopamine Changes in Accumbens from Glucose Intake

**DOI:** 10.1371/journal.pone.0101585

**Published:** 2014-07-07

**Authors:** Gene-Jack Wang, Dardo Tomasi, Antonio Convit, Jean Logan, Christopher T. Wong, Elena Shumay, Joanna S. Fowler, Nora D. Volkow

**Affiliations:** 1 Laboratory of Neuroimaging, National Institute on Alcohol Abuse and Alcoholism, Bethesda, Maryland, United States of America; 2 Department of Psychiatry, New York University, New York, New York, United States of America; 3 Nathan Kline Institute for Psychiatric Research, Orangeburg, New York, United States of America; 4 Biosciences Department, Brookhaven National Laboratory, Upton, New York, United States of America; Duke University Medical Center, United States of America

## Abstract

**Objective:**

Dopamine mediates the rewarding effects of food that can lead to overeating and obesity, which then trigger metabolic neuroadaptations that further perpetuate excessive food consumption. We tested the hypothesis that the dopamine response to calorie intake (independent of palatability) in striatal brain regions is attenuated with increases in weight.

**Method:**

We used positron emission tomography with [^11^C]raclopride to measure dopamine changes triggered by calorie intake by contrasting the effects of an artificial sweetener (sucralose) devoid of calories to that of glucose to assess their association with body mass index (BMI) in nineteen healthy participants (BMI range 21–35).

**Results:**

Neither the measured blood glucose concentrations prior to the sucralose and the glucose challenge days, nor the glucose concentrations following the glucose challenge vary as a function of BMI. In contrast the dopamine changes in ventral striatum (assessed as changes in non-displaceable binding potential of [^11^C]raclopride) triggered by calorie intake (contrast glucose – sucralose) were significantly correlated with BMI (r = 0.68) indicating opposite responses in lean than in obese individuals. Specifically whereas in normal weight individuals (BMI <25) consumption of calories was associated with increases in dopamine in the ventral striatum in obese individuals it was associated with decreases in dopamine.

**Conclusion:**

These findings show reduced dopamine release in ventral striatum with calorie consumption in obese subjects, which might contribute to their excessive food intake to compensate for the deficit between the expected and the actual response to food consumption.

## Introduction

Brain dopamine (DA) modulates eating behaviors through its modulation of reward and incentive salience [1]. Activation of DA in the nucleus accumbens (NAc) occurs with exposure to novel food rewards but with repeated exposures DA increases instead shift to the cues that predict the food reward [2]. The mesolimbic DA system is critical for reinforcing food palatability and highly palatable foods increase DA in NAc [3], whereas DA antagonists attenuate the hedonic value of sucrose [4]. DA also mediates the rewarding effects of food that are driven by energy content [5]. Rodent studies revealed intragastric administration of glucose increased DA in NAc [6], which was an effect dependent on glucose utilization, since administration of an anti-metabolic glucose analog lowered DA. This indicates that DA neurons respond to the energetic value of nutrients independent of taste and implicates postingestive factors in calorie related DA increases in NAc. Moreover in humans neuroimaging studies have shown that sucrose solution but not a non-caloric sweet solution activates the midbrain, which is where DA neurons are located [7]. DA neurons are also activated by visual, auditory and somatosensory stimuli that predict food reward [8]. Excessive food consumption can lead to obesity, which, in turn triggers metabolic adaptations that further perpetuate excessive food consumption. Some of these neuro-adaptations occur in DA pathways as evidenced by clinical and preclinical studies documenting a reduction in DA D2 receptors in striatum with obesity [9].

Here we hypothesized that in obesity the response to calorie consumption would be attenuated just as has been shown for drug consumption in addiction [10]–[12]. For this purpose we used positron emission tomography (PET) and [^11^C]raclopride (D2/D3 receptor radiotracer sensitive to competition with endogenous DA) [13] to assess if calorie induced DA increases in ventral striatum (where NAc is located) are dependent on body mass index (BMI). This is possible because [^11^C]raclopride's binding to D2/D3 receptors is sensitive to the concentration of endogenous DA; such that when DA levels increase the specific binding of [^11^C]raclopride decreases and when DA levels decreases [^11^C]raclopride's specific binding increases [12], [14]. In order to control for the effects of glucose's palatability (sweetness), we contrasted the effects of sucralose (artificial sweetener devoid of calories) to those of glucose. Thus the contrast between the two sweet solutions (one with calories and one without calories) enabled us to measure the changes in DA that are attributable to the calories independent of the food's palatability.

## Methods

This study was carried out at Brookhaven National Laboratory (BNL) and the Stony Brook University's Committee on Research Involving Human Subjects approved the protocol. Written informed consent was obtained from participants prior to study initiation. Nineteen subjects were included in the study if they were right-handed, 40–60 years old, healthy and had 21≤ BMI ≤35 kg/m^2^. Exclusion criteria included history or presence of any medical condition that may alter cerebral function; diabetes mellitus; present or past history of an Axis I diagnosis (including depression or anxiety disorder) as per DSM IV; eating disorders; alcohol or drug abuse or dependence (including nicotine). Subjects were asked to have their last meal completed by 7 PM the evening before the day of the imaging visits and were scanned between 15 and 17 hours after their last meal. Subjects were informed that blood sugar levels would be checked during the study to help ensure that they refrained from eating.

### Study Design

Subjects had two imaging visits: on one day of study (Day A) the subject took a 75 gram oral glucose drink (Trutola, VWR, PA); on the other day (Day B) the subject took an oral placebo drink (sucralose, 0.348 mg/ml [JK Sucralose Inc., NJ] that is of equal volume and sweetness level to the glucose solution). PET started at 10 minutes after completion of the glucose/placebo drink. PET scans were run on a Siemens ECAT HR+ and [^11^C]raclopride was prepared according to methods published previously [15]. Scans were started immediately after tracer injection of 8 mCi or less of [^11^C]raclopride and carried out for a total of 60 minutes. Blood samples for glucose levels were obtained prior to the drinks, immediately upon completion of the glucose/placebo drink, then every 5 minutes for 30 minutes, at 60, 90 & 120 minutes. PET was carried out at the approximate same time of day for all subjects. Subjects were asked to fast and stay hydrated overnight (at least 12 hours) prior to the start of any study procedures on each day of imaging study. Days A and B were randomized across subjects. These two scan days were separated between 2–42 days with an average of 16±10 days.

### Clinical Scales

The eating behavior questionnaires were obtained during the screening visit using the Three Factors Eating Questionnaire-Eating Inventory (TFEQ-EI) to assess the following three dimensions of eating behavior: cognitive processes; behavioral adaptation; and control and the Gormally Binge Eating Disorder Scale (GBEDS) to look at binge eating behavior and associated psychopathology [16]. To assess the palatability of the glucose and sucralose drinks, the subjects were asked to assess quality of sweetness, sweetness level and likeness of the sweetness using self-reports [rated from 1 (less) to 10 (most)] immediately after they consumed the drinks. Linear regression analysis was used to analyze the association between these self-reports and BMI. Pair t-tests were used to compare differences in these self-reports between the glucose and sucralose drinks.

### Blood Glucose Concentration Measurement

Plasma samples were analyzed for glucose concentration using a Beckman Glucose Analyzer 2 (Brea, California), which determines glucose by means of the oxygen rate method employing a Beckman oxygen electrode. A measured volume of sample is pipetted into an enzyme reagent in a cup containing an electrode that responds to and reports oxygen concentration in mg of glucose/100 mL. Paired t-tests were used to analyze the differences in blood glucose levels, independently for each time point. Linear regression analysis was used to assess the association between blood glucose level and BMI.

### Data Analysis

The time-activity curves for tissue concentration in striatum and in cerebellum along with the time activity curves for [^11^C]raclopride were used to calculate the distribution volume (DV) in the pixels for the whole image. Specifically, we estimated for each voxel the DV, which corresponds to the equilibrium measurement of the ratio of the radiotracer's tissue concentration to that of its plasma concentration using a graphical analysis technique for reversible systems [17]. A custom Montreal Neurological Institute template, which we previously developed using the distribution volume images from 34 healthy subjects acquired with [^11^C]raclopride and the same scanning sequence, was used for the spatial normalization of the DV images. For the binding potential (BP_ND_) images we normalized the DV in each voxel to that in the cerebellum (left and right regions-of-interest), which correspond to dopamine (DA) D2/D3 receptor availability [17]. The BP_ND_ images were then spatially smoothed using an 8-mm Gaussian kernel to minimize the variability of the brain anatomy across subjects. Differences in BP_ND_ between glucose and sucralose were used to estimate changes in DA triggered by calories.

### Statistical Analyses

Multilinear regression analysis was used to analyze the association between BP_ND_ differences between the glucose and the sucralose (**Δ**BP_ND_), which reflect changes in DA secondary to the calorie content of glucose. The Statistical Parametric Mapping (SPM8; Wellcome Trust Centre for Neuroimaging, London, UK) was used for this purpose. Statistical significance was set as P_FWE_ <0.05, corrected for multiple comparisons at the voxel level with a familywise error and small volume corrections within a 10-mm radius spherical region-of-interest (ROI). Follow up analyses were conducted on average ROI measures that were extracted using the coordinates obtained from SPM to assess the effect of behavioral measures (comprising scores of cognitive restraint of eating, disinhibition and hunger using TFEQ-EI and the binge eating score using GBEDS), blood glucose levels, age, and gender. Specifically, these variables were correlated with the average **Δ**BP_ND_ signals in the ROI after controlling by BMI. Statistical significance for these correlation analyses was set as P<0.05, uncorrected.

## Results

**Figure 1:**
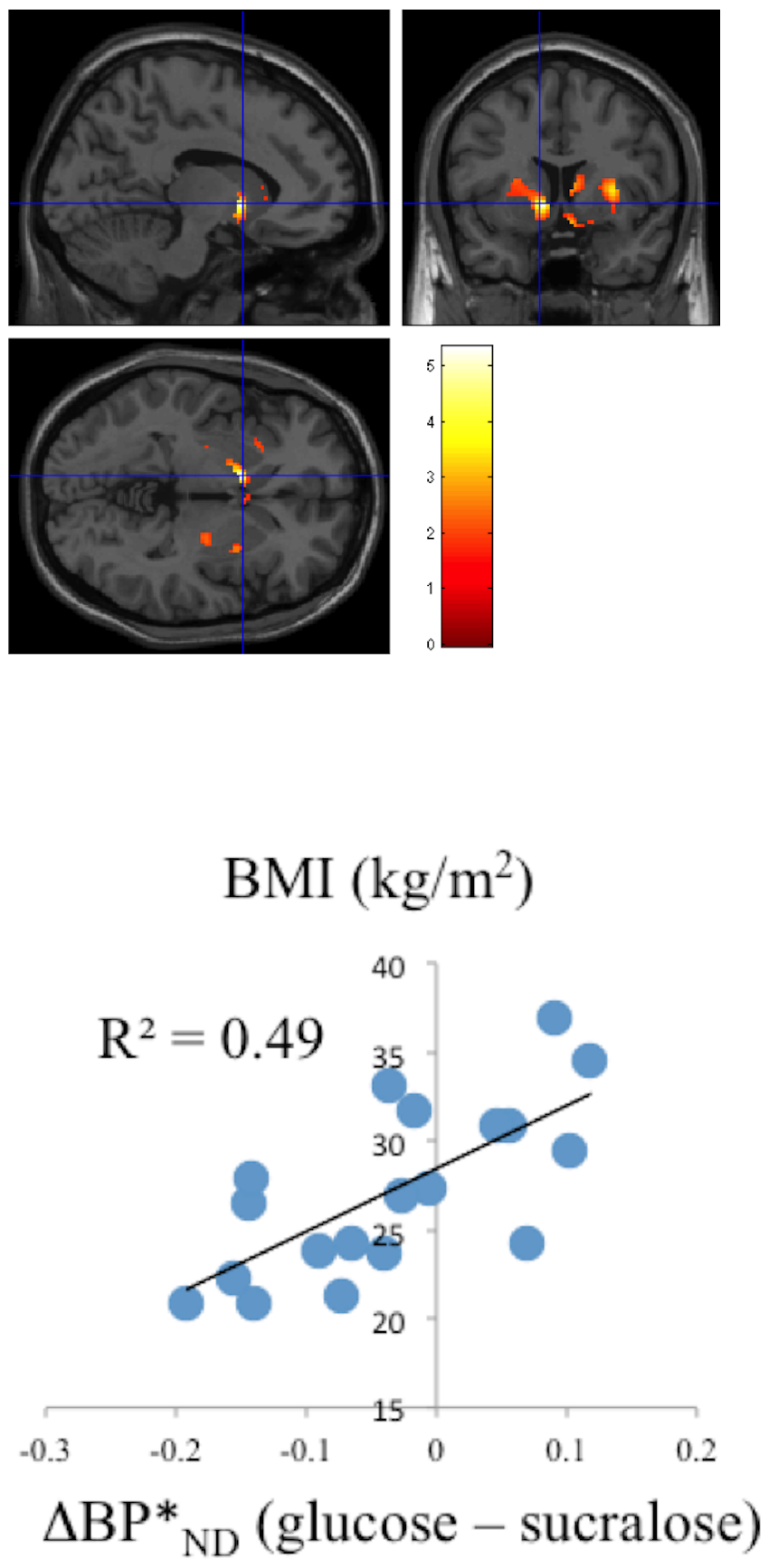
a: SPM images of brain dopamine changes. Significant activated clusters show dopamine (DA) changes in nucleus accumbens for the contrast glucose > sucralose intake (**Δ**BP_ND_). Note that increases in BP_ND_ reflect DA decreases (less competition from DA for [^11^C]raclopride to bind to D2/D3 receptors) whereas decreases in BP_ND_ reflect DA increases with glucose (as compared to sucralose) The SPM images were superimposed onto T2 weighted MR images in sagittal (left upper), coronal (right upper) and transverse (lower) views. The color bar indicates *t*-score values. **b: Correlation between BMI and brain DA changes.** The differences between DRD2 availability after the glucose and the sucralose intake (**Δ**BP_ND_) were compared with BMI (kg/m^2^). The leaner subjects showed the largest DRD2 decreases with glucose in nucleus accumbens (consistent with DA increases) whereas the heavier subjects showed DRD2 increases (consistent with DA decreases). **Δ**BP*: corrected for changes in blood glucose levels (glucose – sucralose) within PET acquisition (0–60min).

The difference of blood glucose concentration did not vary as a function of BMI after the sucralose and glucose challenge (r<0.18, R^2^<0.03). There were no differences between the glucose and sucralose drinks on self-reports for quality of sweetness (glucose: 5.4±2.6. sucralose: 5.4±2.6); sweetness level (glucose: 6.8±2.5. sucralose: 6.2±2.5) and likeness of the sweetness (glucose: 4.7±2.8. sucralose: 4.8±3.0) and these self-reports were not influenced by the subject's BMI. In contrast we observed a significant correlation between calorie-induced DA changes as assessed by **Δ**BP_ND_ (glucose – sucralose) in the ventral striatum (r = 0.68; P_FWE <0.004, P_FDR <0.05, voxels = 131, Fig. 1a) and BMI, such that the lower the BMI the greater the DA increases and the higher the BMI the greater the DA decreases in ventral striatum. The correlation remained significant after covaring with difference of blood glucose concentration (glucose – sucralose) (Fig.1b).

DA changes in response to caloric intake (**Δ**BP_ND_) were also significantly correlated with scores on the eating behavioral measures. Specifically, delta BP_ND_ in ventral striatum was significantly correlated with the measures of eating behaviors the TEFQ-EI scores of disinhibition (r = 0.52, p<0.02) and hunger (r = 0.6, p<0.006) and the GBES scores of binge eating (r = 0.61, p<0.006), such that subjects with greater scores on disinhibition, perception of hunger and binge eating showed decreases in DA with caloric intake. However, these correlations were not significant after covaring for BMI and sex.

## Discussion

In this study, contrasting glucose with sucralose allowed us to assess the effects of calorie consumption in striatal DA signaling after controlling for reward responses associated with palatability. Thus the DA changes in the ventral striatum from this contrast reflect responses from the energy content of glucose consumption. The opposite patterns of DA responses in the ventral striatum in lean individuals who showed DA increases in contrast to the DA decreases observed in obese subjects, might reflect the differences between the expected and the actual response to caloric intake since DA responses are influenced by reward probability distributions [18]. Specifically a reward that is better than predicted elicits an activation of DA neurons and a reward that is worse than predicted induces inhibition [19]. Even though glucose concentrations in blood were similar between lean and obese subjects, the response to the caloric content in the obese subjects would have resulted in less than the predicted response resulting in inhibition of DA neurons and reduced DA release after the glucose drink. However because we did not obtain measures of D2/D3 receptor availability without the administration of a sweetened solution (baseline measure) we cannot rule out the possibility that the abnormal response in the obese subject is also driven by an abnormal response to sweetness and not just an abnormal response to calories.

In mice lacking functional sweet taste receptors sucrose but not an artificial sweetener increased DA in NAc [20], which is consistent with our findings showing DA increases in ventral striatum triggered by caloric ingestion in lean individuals. However such a response was not observed in obese individuals indicating a disruption of the brain DA responses to caloric content.

Greater scores on TEFQ disinhibition are associated with impaired control of food intake [21] and have been linked with worse frontal executive function [21], [22]. They are also consistent with our prior findings showing a significant correlation between food restraint scores and striatal DA increases induced by exposure to food cues [23], thus supporting an association between decreased striatal DA signaling and impaired self-control [24]. The correlation of hunger on the TFEQ with DA change in NAc with caloric provides further evidence for DA's role in hunger perception in humans [25]. Finally the association between decreases in DA after glucose and greater binge eating scores is reminiscent of the decreases in stimulants induced DA increases in cocaine abusers whose behavior is characterized by compulsive cocaine intake [10], [12], [26]. Though it is tempting to invoke a hypo-responsivity of the DA reward circuit in obese subjects this is an inadequate descriptor; for we specifically observed a hypo-responsivity to the calorie consumption but it is plausible that they might have hyper-responsivity to exposure to food cues. It is therefore more likely that a discrepancy between an enhanced expectation and a reduced response to the calories consumed in the obese person might trigger the drive to continue eating in order to compensate for this deficit.
